# Development and reliability testing of a self-report instrument to measure the office layout as a correlate of occupational sitting

**DOI:** 10.1186/1479-5868-10-16

**Published:** 2013-02-04

**Authors:** Mitch J Duncan, Mahbub Rashid, Corneel Vandelanotte, Nicoleta Cutumisu, Ronald C Plotnikoff

**Affiliations:** 1 Central Queensland University, Institute for Health and Social Science Research, Centre for Physical Activity Studies, Bld 18, 4702, Rockhampton, Queensland, Australia; 2 Department of Architecture, School of Architecture, Design, and Planning, University of Kansas, 66045, Lawrence, Kansas, USA; 3 Sedentary Living Laboratory, and the Alberta Centre for Active Living, Faculty of Physical Education and Recreation, University of Alberta, T6G 2H9, Edmonton, Alberta, Canada; 4 Priority Research Centre in Physical Activity and Nutrition, University of Newcastle, 2308, Callaghan, New South Wales, Australia

**Keywords:** Sitting, Correlates, Workplace, Environment, Spatial configuration

## Abstract

**Background:**

Spatial configurations of office environments assessed by Space Syntax methodologies are related to employee movement patterns. These methods require analysis of floors plans which are not readily available in large population-based studies or otherwise unavailable. Therefore a self-report instrument to assess spatial configurations of office environments using four scales was developed.

**Methods:**

The scales are: local connectivity (16 items), overall connectivity (11 items), visibility of co-workers (10 items), and proximity of co-workers (5 items). A panel cohort (N = 1154) completed an online survey, only data from individuals employed in office-based occupations (n = 307) were used to assess scale measurement properties. To assess test-retest reliability a separate sample of 37 office-based workers completed the survey on two occasions 7.7 (±3.2) days apart. Redundant scale items were eliminated using factor analysis; Chronbach’s α was used to evaluate internal consistency and test re-test reliability (retest-ICC). ANOVA was employed to examine differences between office types (Private, Shared, Open) as a measure of construct validity. Generalized Linear Models were used to examine relationships between spatial configuration scales and the duration of and frequency of breaks in occupational sitting.

**Results:**

The number of items on all scales were reduced, Chronbach’s α and ICCs indicated good scale internal consistency and test re-test reliability: local connectivity (5 items; α = 0.70; retest-ICC = 0.84), overall connectivity (6 items; α = 0.86; retest-ICC = 0.87), visibility of co-workers (4 items; α = 0.78; retest-ICC = 0.86), and proximity of co-workers (3 items; α = 0.85; retest-ICC = 0.70). Significant (p ≤ 0.001) differences, in theoretically expected directions, were observed for all scales between office types, except overall connectivity. Significant associations were observed between all scales and occupational sitting behaviour (p ≤ 0.05).

**Conclusion:**

All scales have good measurement properties indicating the instrument may be a useful alternative to Space Syntax to examine environmental correlates of occupational sitting in population surveys.

## Background

Individuals employed in office-based occupations typically engage in high levels of occupational sitting. Modification of office environments is one strategy in a multilevel intervention approach that can be used to prevent long bouts of sitting and reduce overall sitting time, and in doing so reduce the ill health effects of prolonged sitting such as increased risk of obesity, diabetes, CVD and premature mortality [1-5]. However, applying a multilevel intervention approach requires knowledge of how characteristics at the multiple intervention levels influence sitting behaviours, and existing literature examining correlates of occupational sitting is dominated by individual-level correlates [6,7]. Despite this, methods exist to objectively quantify and characterise office environments to then examine how office environments influence walking behaviours and stair usage [8-11], yet the relationship of office environments and sitting behaviour is seldom explored [8].

Objective quantification of office environments can be conducted using analytic techniques such as Space Syntax which can characterise the shape of office environments, and in particular the way in which rooms, workstations, office furniture and walkways are located in the office environment [8-10,12]. Space Syntax frequently uses the axial map analysis technique to examine these settings. The axial map of a setting is composed of the minimum number of straight lines (or lines of sight), also known as axial lines, needed to cover every space and circulation rings of the setting [9,13,14]. The two main outcomes of this analysis, connectivity and integration, have been used to describe the relationships between the movement patterns of individuals and spatial layouts of office environments [9,14]. The connectivity value of an axial line represents the amount of potential alternative movement routes available to a person from that axial line to other axial lines that intersect it. The integration value of an axial line expresses how accessible an axial line is from all the other axial lines present in the axial map of the setting or the global accessibility of the floor plan.

Space Syntax studies may also use measures of the proximity and the visibility of co-workers as correlates of movement in office environments to supplement measures of connectivity and integration. For example, whilst integration is a good measure of network or global accessibility, proximity of co-workers is a good measure of their physical closeness in workplaces. Proximity of co-workers is measured as the mean length of axial lines per workspace in an office, thus the lower the mean length of axial lines the greater the proximity of workspaces and co-workers. Co-worker visibility can be measured using either visual field analysis or Visibility Graph Analysis (VGA) [8]. Visual field analysis employs the visual field (defined as a 360-degree polygon) as the unit of analysis; and higher visibility is defined as a higher number of co-workers or workstations within a visual field [8,9]. In contrast, VGA involves the creation of a graph of mutually-visible locations in a spatial system and a higher number of mutually-visible locations represent higher visibility [15].

As spatial configuration characteristics of office settings are associated with increased occupant movement, the measures of Integration, Connectivity, Proximity of Co-workers and Visibility of Co-workers may provide insight on how to modify environments to reduce occupational sitting. For example, increased connectivity and integration are associated with higher levels of walking for any purpose in a building [8,10], and the frequency of unscheduled office visits between co-workers is positively associated with increased levels of integration in the workplace [16]. It has also been demonstrated that more frequent face-to-face interactions between co-workers occur in office settings where the proximity and visibility of co-workers is greater [9,10,16-18]. While positive relationships have been observed between co-worker proximity and face-to-face interactions [19], it is also possible proximity may also reduce movement and increase sitting when co-workers communicate without leaving their workstations when they are very proximal to each other or employees perceive a need to be at their workstation [8,12]. Holding all other things equal, individuals in buildings that display greater connectivity, integration, visibility, and proximity may walk more and sit less in the office setting compared to individuals in buildings at the opposite end of this spatial configuration continuum. Yet office types are not uniform, and different office types display different spatial configurations as defined by Space Syntax. For example, the typical Open Plan Office has greater levels of connectivity and integration compared to the typical office dominated by private single occupant offices [12], therefore office type is also important to consider when examining spatial configurations of office settings.

While characteristics of office configurations can be quantified using Space Syntax-based measures, these methods are limited by the requirement to obtain detailed floor plans for analysis. This has meant that studies of this type have been limited to either small populations (< 100 people) or few buildings [9,10]. This limits the generalizability of findings and makes it difficult to apply these techniques in larger samples to understand the relationships between office environments and sitting in larger and more diverse populations. Despite this limitation, Space Syntax and related methods are useful during the design or redesign phase as architects and designers can apply these techniques to a proposed design and modify it to manipulate different levels of connectivity and co-worker visibility and proximity. The utility of this approach is demonstrated by studies that examine the configuration of office environments before and after the redesign phase and document improvements in connectivity related measures and co-worker visibility [10]. As such, we sought to develop a self-report instrument that would capture underlying constructs similar to those available from Space Syntax that was brief enough to be used in population-based surveys, and describe the psychometric properties of the instrument.

## Methods

The aims of the current study are achieved in two phases. Phase one of the study describes the development and refinement of the instrument, which was named the Office Environment and Sitting Scale (OFFESS), whereas phase two examines the test-retest reliability of the OFFESS. Occupational sitting behaviour was assessed in both phases using two items developed specifically for this study, one measuring the duration of sitting and one measuring the number of breaks in sitting. Information on the psychometric properties of these items is reported in both phase one and two of this study.

### Phase one

#### OFFESS instrument development

The OFFESS includes scales that reflect the Space Syntax constructs of connectivity, integration, proximity of co-workers, and visibility of co-workers. A single item to measure the type of office environment was included as spatial configuration measures are not consistent across office types [12].

Connectivity and integration measures are closely related but they represent different characteristics of building floor plans that are important to movement, therefore two separate scales, “Local Connectivity” and “Overall Connectivity”, were developed to represent connectivity and integration, respectively. During the development of items for these scales, it was necessary to establish items that were meaningful and interpretable to respondents while remaining consistent with the underlying Space Syntax constructs; therefore, easily interpretable terminology, such as ‘lines of sight’ and ‘passageways or walkways’ were used to represent axial lines. The preliminary Local Connectivity scale comprised 16 items and the preliminary Overall Connectivity scale comprised 11 items, items can be viewed in Table 1. The preliminary Co-worker Visibility scale, centred on the number of colleagues and workstations that an individual could visually observe from their own workstation, included 10 items. The preliminary Co-worker Proximity scale included 5 items that focused on the perceived proximity of multiple colleagues and being able to hear quiet noises from colleague’s workstations as measures of physical proximity (Table 1). The Local Connectivity, Overall Connectivity, Visibility of Co-worker and Proximity of Co-worker scales were answered using 4 point Likert Scale ranging from Strongly Agree to Strongly Disagree; each scale is scored as an average of item responses. A 4 point Likert Scale has been used in previous questionnaires examining perceptions of the environment [20,21].

**Table 1:** Description of items included in the original and the refined scales of local connectivity, overall connectivity, proximity of co-workers, visibility of co-workers

	**Included in refined scale**	**Mean (± SD)**^**1**^
**Local Connectivity**		
Hallways and passageways in my building frequently intersect each other	Yes	2.35 (0.88)
There are many long hallways and passageways in my building		2.22 (0.84)
To get to the hallway/passageway closest to my workstation/desk I need to make many changes in direction		1.84 (0.85)
When travelling along hallways and passageways you can enter common areas such as kitchens, foyers and coffee rooms with a single change in direction		2.75 (0.85)
When standing in common areas such as kitchens/meeting rooms you can see into many other rooms		1.99 (0.78)
Office doors and other partitions between sections of the building are always closed		2.00 (0.80)
When standing at my workstation/desk I can see into many other rooms		2.05 (0.78)
Desks/workstations are located on either side of hallways and passageways		2.42 (0.89)
There are many direct "lines of sight" between workstations/desks		2.47 (0.87)
There are many alternative routes to move around my office (I don't have to go the same way every time)	Yes	2.25 (0.91)
Clearly defined pathways for travel between workstations frequently intersect with each other	Yes	2.34 (0.80)
I can access kitchen or coffee rooms directly from hallways/passageways	Yes	3.28 (0.74)
I can take many different travel routes through the office to reach the same destination when travelling	Yes	2.24 (0.95)
Passageways are well defined		3.10 (0.70)
There are few straight hallways and passageways		2.34 (0.85)
To access most of the separate offices (offices enclosed by floor to ceiling walls) in the building I have to move through another office first (ie a receptionist or administrators office)		1.93 (0.92)
**Overall Connectivity**		
I can access most offices in the building directly from hallways/passageways without passing through another office or room		2.92 (0.90)
My office building has many rooms that are difficult to find	Yes	1.64 (0.74)
In my office building you have to pass through at least one other office/room to reach most offices		1.89 (0.85)
I can access toilets directly from hallways/passageways		3.25 (0.81)
I can walk to colleagues desks/workstations easily without many changes in direction		2.93 (0.74)
Walking in my building requires frequent changes in direction one after another	Yes	2.04 (0.74)
To travel from my workstation/desk to the closest toilet requires many changes in direction	Yes	2.06 (0.78)
To travel from my workstation/desk to the closest meeting room/area requires many changes in direction	Yes	2.01 (0.73)
To travel from the main entry of my building/floor to my workstation/desk requires many changes in direction	Yes	2.09 (0.76)
I can walk to the workstation/desk of a co-worker without making any changes in direction		2.93 (0.74)
Walking from my own workstation/desk to most others in the building requires many changes in direction	Yes	2.15 (0.79)
**Proximity of Co -Workers**		
From my workstation/desk I can hear other people talking quietly at their workstation/desk	Yes	2.92 (0.85)
There are many other workstations/desks located in my building within a short walk of my workstation/desk	Yes	2.88 (0.84)
In the area surrounding my workstation/desk there are lots of other workstations/desks	Yes	2.50 (0.92)
The distance between my workstation/desk and the workstation/desk of co-workers I need to communicate with for my work makes it easier to call or email them than walk to their workstation/desk		1.95 (0.82)
There are many other workstations/desks located within a 1 minute walk of my workstation/desk		2.98 (0.90)
**Visibility of Co-workers**		
From my workstation/desk I can see several colleagues sitting or standing at their workstations/desks (do not include offices with the door closed)	Yes	2.59 (1.20)
From my workstation/desk I can see several colleagues closed office doors		1.58 (0.79)
I frequently "bump in to" other people when walking in my building	Yes	2.90 (1.01)
I cannot actually see my colleagues at their desk because of physical barriers such as walls, high partitions and closed doors		2.15 (1.16)
Colleagues regularly see me at my workstation/desk and either wave, say hello, or stop for a conversation		2.90 (0.90)
When walking down hallways in my building I can see into areas like kitchens, coffee rooms, mail rooms, meeting rooms		2.97 (0.91)
There are many entry points to reach the floor that I work on within my building (do not consider locked fire exits)		2.19 (0.98)
I frequently see people/other employees walking around inside the building	Yes	3.01 (0.84)
I frequently see people/other employees standing and talking inside the building	Yes	2.89 (0.90)
When walking in hallways near my desk/workstation, I can see my desk/workstation from the hallway		2.49 (1.01)

Note. The absence of “Yes” beside an item indicates it was excluded from the refined scale. 1 – data are based on the means (SD) of the original items in the Phase one sample.

Connectivity and integration have shown to differ between Open Plan Offices and Private Enclosed Offices [12], but other office types, such as private shared offices and open-plan offices with either high or low partitions between workstations, exist [22]. Private Shared Offices are those with up to approximately four workstations in the office, and low and high dividers between workstations in Open Plan Offices are typically those less than or greater than 1.5 m, respectively. A single item was used to assess office type: “Now please think about the area where most desks or workstations are located. Which of the following best describes the location of the majority of desks/workstations in your building?” Response options were:

1. In an office separated from other offices by floor to ceiling walls, door, not shared with anyone else,

2. In an office separated from other offices by floor to ceiling walls, door, shared by 2–4 people,

3. In a single area containing many desks/workstations separated by high partitions (greater than 1.5 m (5 feet) in height),

4. In a single area containing many desks/workstations separated by low partitions (less than 1.5 m (5 feet) in height),

5. In a single area containing many desks/workstations separated by no partitions.

For the purposes of the current study, office type was collapsed into three categories: Private Enclosed Offices (1), Shared Offices (2 and 3) and Open Plan Offices (4 and 5).

All items were initially developed by the lead author (MJD) based on literature on the measurement and analysis of spatial configurations of locations [8-10,12,23,24]. Clarification of wording and ambiguous terminology in items was conducted following a review by two of the authors (MR, NC) with knowledge of architectural, human behaviour theory, and experience in applying Space Syntax to examine the spatial configuration of locations.

#### Participants and procedures

Participants were members of the Australian Health and Social Science (AHSS) Panel (n = 1609), a random sample of Australian adults (aged 18 years and older) who reside in all States and Territories of Australia [25]. The AHSS Panel completes regular online surveys on various issues related to physical and mental wellbeing, and health outcomes. Details of the sample recruitment are provided elsewhere [25], a brief summary is provided here. The AHSS Panel members were recruited via a brief CATI survey performed by the Population Research Laboratory at CQUniversity. Telephone numbers were drawn from listings of land line telephone numbers held by the Population Research Laboratory that were able to be direct-dialled. Following recruitment into the AHSS Panel, participants were emailed an individually-addressed invitation to take part in an online survey to collect information on socio-demographic details, health outcomes, and behaviours. The current survey was conducted in February to March 2010. Panel members who had not completed the survey were sent up to five email reminders to invite them to complete the survey. The response rate to this online survey was 54% and compares favourably to other recently conducted online surveys [26].

#### Survey instrument

Participants provided information on socio-demographic characteristics, dietary habits, sleep habits [27], office environment, preliminary OFFESS items, job autonomy [28], health outcomes (presence of chronic disease, physical activity limitation), health-related quality of life [29], physical activity (IPAQ-LF) [30], and the presence, frequency of travel, and distance from the workstation of 12 destinations in the workplace. Sitting behaviours in leisure time was assessed using items based on previously-developed instruments [31]. An existing measure of the duration of occupational sitting was modified for the current study to explicitly exclude sitting in a motor vehicle [32,33]. The wording of the modified item was “How much time do you usually spend sitting when at work only. This may include the time you spent sitting while at your desk, sitting during meetings, and at lunch. Do not include the time you spent travelling in a motor vehicle during work hours”. The frequency of breaks in occupational sitting was assessed using the following item “During the last 7 days, approximately how many times per day did you get up from your desk or workstation/area for longer than one minute? This may include things like going to the printer or utility room, bathroom, walking to a meeting, walking to the kitchen/lunch area, or a colleague’s desk.” Only information related to the office environment and sitting behaviours are reported in phase one.

#### Socio-demographics

Participants provided details on their age, gender, household income, highest level of educational attainment, BMI, number of years of education, hours of work, occupational status and level. The setting of work undertaken was assessed using a single item “Which of the following best describes the location of your employment?” response options were office-based, workshop-based, outdoors, combinations of these settings, and other. The type of work undertaken by participants while at work was determined using a single item “On the days that you work, what would you consider to be the main activity that you perform” with the following response options: administration and/or reception, computer, desk work or reading, customer service and/or retail activities, manufacturing, physically demanding work (tradesperson). This item was based on an item used to classify occupational activity levels [34].

### Phase two

#### Participants and procedures

Participants in phase two were recruited by an email to staff at a single campus of a regional University inviting participation in the study. Participants were informed that they would be required to complete the survey on two separate occasions one week apart. Sixty-three individuals completed the first administration. Only those participants who completed the first survey were invited to complete the second administration of the survey; forty-three participants completed the second administration of the survey. Non responders to the second survey were prompted via email up to five times to complete the survey.

#### Survey instrument

The same survey instrument from phase one was administered to phase two participants. Phase two reports on items related to office environment and the two occupational sitting behaviour items.

### Statistical analysis

#### Phase one

As the aim of the study was to evaluate the psycho-metric properties of an instrument designed to assess office environments, analysis of the AHSS Panel data was delimited to: those who were employed full-time or part-time; the primary setting of their work was an office; and, those engaged in administration, reception, computer or desk-based work activities (n = 307). The number of participants included in the analysis in phase one is sufficient for the purposes of the study [35]. Consistent with the aims of phase one, factor analysis with principal components extraction and varimax rotation was used to identify redundant items (eigenvalues <0.30) within each OFFESS scale. Additionally, a parsimonious approach was taken to retain items considered to be theoretically important in representing underlying Space Syntax constructs; this was conducted by two of the authors (MJD, MR). Confirmatory factor analysis was then conducted to confirm a single factor structure of the refined scale. Following this process, the internal consistency of the original scale and the refined scale was evaluated using Cronbach’s alpha (α); the internal consistency was considered acceptable when α was greater than 0.70 [36]. In the absence of direct comparison to Space Syntax derived variables, the validity of the OFFESS was assessed by examining correlations between scales (Pearson Product Moment Correlations), differences in scales between office types (ANOVA), and associations between OFFESS and the duration and frequency of breaks in occupational sitting (Generalized Linear Models: duration of occupational sitting (Linear model with identity link), frequency of breaks in occupational sitting (Poisson model with Log Link)). Associations between OFFESS and the duration and frequency of breaks in occupational sitting are presented for all office types and also stratified by office type as significant interactions were observed between OFFESS constructs and office type for both outcomes (p ≤ 0.05). Covariates used in Generalized Linear Model analyses are stated in the notes section of Table 2. Correlations between the duration of occupational sitting time item used in this study, the frequency of breaks in occupational sitting, and the IPAQ-LF measured duration of weekday sitting were examined using Spearman’s Rho. Spearman’s Rho correlations were also examined between the duration of occupational sitting time item and the frequency of breaks in occupational sitting. All analyses were conducted using PASW version 18 using a p value of 0.05.

**Table 2:** Relationships between refined OFFESS scales, duration and frequency of breaks in occupational sitting in the phase one sample

	**Scale**			
	**Local connectivity**	**Overall connectivity**	**Co-worker visibility**	**Co-worker proximity**
	***b *(95% CI)**	***b *(95% CI)**	***b *(95% CI)**	***b *(95% CI)**
**Occupational Sitting (mins /day)**				
Total Sample^1^	0.45 (−21.73- 22.63)	−10.03 (−30.71- 10.65)	−6.87 (−26.97- 13.24)	23.26 (4.08-42.45)*
Office Type				
Private Enclosed Office (n = 70)^**2**^	−20.66 (−76.30- 34.78)	54.25 (8.84- 99.66)*	−35.82 (−85.36- 13.72)	26.25 (−20.14- 72.64)
Shared Office (n = 59)^**2**^	−41.06 (−107.08- 24.95)	−17.34 (−77.85- 43.16)	20.14 (−26.62- 66.89)	21.03 (−28.30- 70.36)
Open Plan Office (n = 172)^**2**^	4.67 (−21.00- 30.32)	−27.80 (−53.10- -2.50)*	−2.67 (−27.34- 22.00)	28.91 (6.22- 51.60)*
**Breaks in Sitting (times /day)**				
Total Sample^3^	−0.02 (−0.08- 0.034)	−0.11 (−0.17- -0.06)*	0.15 (0.09- 0.20)*	0.02 (−0.03- 0.07)
Office Type				
Private Enclosed Office (n = 70)^**4**^	0.47 (0.34- 0.60)*	−0.34 (−0.45- -0.23)*	−0.19 (−0.21- -0.08)*	0.15 (0.04- 0.26)*
Shared Office (n = 59)^**4**^	0.28 (0.09- 0.47)*	−0.19 (−0.35- -0.02)*	0.10 (−0.03- 0.23)	0.02 (−0.11- 0.15)
Open Plan Office (n = 172)^**4**^	−0.22 (−0.29- -0.15)*	−0.03 (−0.10- 0.05)	0.30 (0.22- 0.37)*	0.01 (−0.05- 0.08)

Notes: *p = <0.05. 1. Covariates included in the analysis – gender, occupational category, autonomy, BMI, age, and office type. 2. Covariates included in the analysis – gender, occupational category, autonomy, BMI, and age. 3. Covariates included in the analysis – gender, occupational category, autonomy, BMI, age, office type, and duration of occupational sitting. 4. Covariates included in the analysis – gender, occupational category, autonomy, BMI, age, and duration of occupational sitting.

#### Phase two

Similar to phase one, only full-time or part-time employed office based workers whose primary job tasks were based at a workstation were included in the analysis (n = 37). The included sample provides adequate sample size for the analysis of test-retest reliability of the OFFESS and the sitting behaviour items in the current study [37]. Internal consistency of the OFFESS was also evaluated in the phase two sample using data from the first survey administration to confirm the structure of the scales in a separate sample. Intraclass Correlation Coefficients and kappa statistics were used to assess the test-retest reliability of continuous and nominal variables respectively, and were interpreted using the following criteria: poor <0.00; slight 0.00-0.20; fair 0.21-0.40; moderate 0.41-0.60; substantial 0.61-0.80; excellent 0.81-1.00 [38].

## Results

### Phase one

Of the 1609 AHSS Panel Cohort members invited to participate in the study, 1154 provided complete data and 307 of these satisfied inclusion criteria for subsequent analysis. Of those individuals excluded from analysis, 202 were retired, 339 did not work in an office setting and 114 worked in retail or customer service forms of employment and were excluded on these individual criteria alone. A description of the socio-demographic individuals who satisfied the inclusion criteria is provided in Table 3. The average age of these participants was 48.74 (SD ±10.14), a higher proportion was female (53.1%), the majority (67.1%) of the sample had a university degree and worked in Open Plan Offices (57.0%). The average time spent sitting at work was 382.79 (SD ±105.03), and the average number of breaks per day was 15.76 (SD ±13.69). The duration of sitting time was inversely associated with the frequency of breaks in occupational sitting (Spearman’s Rho = −0.18, p = <0.001) and positively associated with weekday sitting time measured by the IPAQ-LF (Spearman’s Rho = 0.55, p = <0.001).

**Table 3:** Description of socio-demographic, behavioural and environmental characteristics of the phase one and phase two samples

	**Phase one**	**Phase two**
	**n (%), mean ± SD, median (1**^**st **^**to 3**^**rd **^**quartile)**	**n (%), mean ± SD, median (1**^**st **^**to 3**^**rd **^**quartile)**
**N**	**307**	**37**
**Gender (n %)**		
Male	144 (46.9 %)	9 (24.3 %)
Female	163 (53.1 %)	27 (75.7 %)
**Age (mean ± SD)**	48.74 (10.14)	40.92 (10.99)
**BMI (mean ± SD)**	27.40 (5.82)	27.42 (6.69) ^1^
**Highest Level of Education (n %)**		
Secondary School or less	47 (15.3 %)	0 (0.0 %)
Technical or Trade Certificate	54 (17.6 %)	5 (13.5 %)
University Degree	206 (67.1 %)	32 (86.5 %)
**Occupational Classification (n %)**		
Professional	244 (80.0)	26 (70.3 %)
White Collar	61 (20.0)	11 (29.7 %)
**Occupational Sitting (mins /day) (mean ± SD)**	382.79 (105.03)	399.32 (64.84)
**Breaks in Sitting (times /day) (median 1**^**st**^**,3**^**rd **^**quartile)**	10 (8,20)	10 (5.25, 15)
**Office Type (n %)**		
Private Enclosed Office	71 (23.1 %)	20 (54.1 %)
Shared Office	61 (19.9 %)	9 (24.3 %)
Open Plan Office	175 (57.0 %)	8 (21.6 %)

Note ^1^ – due to missing data BMI is reported for 35 individuals.

The average scores and the internal consistency scores for each of the preliminary scales are displayed in Table 4. Examination of the internal consistency scores reveals that the internal consistency for the preliminary scales of Local Connectivity (α = 0.67) and Overall Connectivity (α = 0.62) were not acceptable and that the internal consistency of the preliminary scales of Proximity of Co-workers (α = 0.77) and Visibility of Co-workers (α = 0.73) were acceptable. Following the factor analysis process to refine the scales, the level of internal consistency for each scale increased to a level considered to be acceptable (α ≥ 0.70) and the number of items per scale was reduced (Table 4). The correlations between scales were all statistically significant (p = 0.01) and of a small to moderate magnitude (Table 5). When examined by office type, the refined OFFESS scales of Local Connectivity, Proximity of Co-workers, and Visibility of Co-workers displayed significant differences by office type (p ≤ 0.05); the Overall Connectivity scale did not differ by office type (p ≥ 0.05) (Table 6).

**Table 4:** Summary of average scores, internal consistency and test-retest reliability for OFFESS scales

	**Phase one sample (n = 307)**						**Phase two sample (n = 37)**		
	**Preliminary scale**			**Refined scale**			**Refined scale**		
	**Items**	**α**	**M (SD)**	**Items**	**α**	**M (SD)**	**α**	**M (SD)**	**ICC**
Local Connectivity	16	0.67	2.35 (0.34)	5	0.70	2.29 (0.58)	0.61	2.30 (0.27)	0.84
Overall Connectivity	11	0.62	2.35 (0.36)	6	0.86	2.49 (0.58)	0.71	2.40 (0.24)	0.87
Proximity of Co-Workers	5	0.77	2.65 (0.62)	3	0.85	2.77 (0.77)	0.46	2.71 (0.48)	0.70
Visibility of Co-Workers	10	0.73	2.57 (0.53)	4	0.78	2.85 (0.77)	0.67	2.94 (0.41)	0.86

**Table 5:** Bivariate correlations between individual scales of the OFFESS (n = 307)

	**Scale**			
**Scale**	**Local connectivity**	**Overall connectivity**	**Co-worker visibility**	**Co-worker proximity**
Local Connectivity	1	0.301**	0.463**	0.337**
Overall Connectivity		1	0.292**	0.220**
Visibility of Co-Workers			1	0.598**
Proximity of Co-workers				1

Notes. ** Indicates p = 0.01.

**Table 6:** Differences in refined OFFESS scales by self-reported office type in the phase one sample

	**Office type**				
	**Private enclosed office (n = 71)**	**Shared office (n = 61)**	**Open plan office (n = 175)**		
	**M (SD)**	**M (SD)**	**M (SD)**	**F**	***p***
Local Connectivity	2.29 (0.58)	2.40 (0.49)	2.61 (0.59)†	9.180	<0.001
Overall Connectedness	1.88 (0.65)	1.98 (0.48)	2.05 (0.58)	2.472	0.086
Visibility of Co-workers	2.36 (0.84)	2.67 (0.71)†	3.10 (0.64)†	30.534	<0.001
Proximity of Co-workers	2.21 (0.78)	2.57 (0.57)†	3.07 (0.66)†	44.526	<0.001

Notes: † = Significantly different to all other office types. p = <0.05.

In the total sample, there was a significant association between the duration of sitting and proximity of co-workers, and there were significant associations between the frequency of breaks in sitting and local connectivity and the visibility of co-workers (p = <0.05) (Table 2). Examination of interaction effects revealed significant interactions between office types, overall connectivity and the duration of occupational sitting (p = <0.05). Significant interactions were observed between office types, all OFFESS constructs and the frequency of breaks in sitting (p = <0.05). To assist in interpreting associations between sitting behaviours and OFFESS constructs, associations are presented stratified by office type (Table 2).

All OFFESS scales were significantly related to the duration of and/or the frequency of breaks in occupational sitting; these relationships varied by office type (Table 2).

### Phase two

An overview of the phase two sample is provided in Table 3. Phase one and two samples were comparable on measures of BMI, minutes of occupational sitting, breaks in sitting time, and occupational classification. However, the phase two sample had a higher proportion of females, people holding university degree qualifications, and people working in Private Enclosed Offices.

The average time between survey completions was 7.7 (SD ±3.2) days. The test-retest of the duration of occupational sitting time item was excellent (ICC = 0.95), whilst the test-retest reliability of the breaks in sitting time (ICC = 0.52) item was moderate. The test-retest reliability of the refined scales of Local Connectivity (ICC = 0.84), Overall Connectivity (ICC = 0.87) and Visibility of Co-workers (ICC = 0.86) scales were excellent, and the test-retest reliability of the Proximity of Co-workers scale (ICC = 0.70) was substantial (Table 4) [38]. The item used to assess office type (Private Enclosed Offices, Shared Office, Open Plan Office) demonstrated substantial levels of agreement between the two survey administrations (κ = 0.73).

## Discussion

Prolonged and uninterrupted sitting is increasingly recognised as a risk factor for ill health. As a result, it is imperative to limit the volume of sitting in populations that engage in high levels of sitting to improve the health of these populations [7,39,40]. White collar and professional occupation groups currently engage in high levels of occupational sitting [33,41,42] and the high level of sitting is likely a function of the social and environmental characteristics of the workplaces these groups occupy [42]. Thus, it is important to understand how these factors influence sitting within the office environment and to do so within behaviour and setting-specific Ecological Models [19,43,44].

This study described the development and measurement properties of a self-report instrument, the OFFESS, to examine spatial characteristics of office environments as potential correlates of occupational sitting. Outcomes of this study suggest that the individual scales of the OFFESS hold acceptable levels of internal consistency and substantial to excellent levels of test-retest reliability. Additionally, it appears that the OFFESS has good construct validity as the individual scales vary by office type in theoretically expected directions and the correlations between individual scales are comparable in magnitude to those observed when using objective measures of Space Syntax [9,12]. Furthermore, significant associations between all OFFESS scales and either one or both of the occupational sitting behaviours were observed in all office types. Significant associations between local connectivity and the frequency of breaks (private enclosed and shared office types), overall connectivity and the duration of sitting (open plan office types), co-worker visibility and the frequency of breaks (open plan offices) and the proximity of co-workers and the frequency of breaks (private enclosed office types) were observed in hypothesised directions. The magnitude of these associations were modest which is consistent with way in which environments are thought to influence activity behaviours - small changes to the behaviours of all people in the environment over long periods of time [44-46]. The associations observed between office spatial configuration and sitting behaviours highlights the need to continue to examine these factors to better understand how to modify occupational sitting behaviours to improve the health of workers.

The internal consistency, test-retest reliability and associations between the OFFESS and occupational sitting behaviour suggest that the OFFESS is a useful instrument to examine spatial configurations of office environments as potential correlates of occupational sitting. However, it must be noted that the direction of several of the significant associations observed were counter to that expected under the hypothesis that greater connectivity (local and proximity) and greater visibility and proximity of colleagues would drive reduced duration of occupational sitting and more frequent breaks in sitting. Examples of the counter expected relationships include associations between frequency of breaks in sitting and local connectivity in open plan offices, sitting duration, break frequency and overall connectivity in private enclosed offices, and sitting duration and co-worker proximity in open plan offices. Potential reasons for these counter intuitive associations may be related to the cultural norms of workplaces or the inherent job characteristics. For example, inverse associations between break frequency and local connectivity in open plan offices could be impacted by employees in these offices minimising breaks to reduce disruptions caused to other employees, a commonly reported annoyance in open plan offices [47]. Alternatively, cultural norms such as the perceived need to be at the workstation in order to be viewed as productive or a mutual surveillance effect may have impacted the associations [8,48]. Workplace norms and policy, towards electronic communication may also impact these associations in workplaces where electronic forms of communication are encouraged for record-keeping purposes, which may be a stronger influence on behaviour than spatial configuration. Cultural norms and communication mode preference and policies were not examined in this study and highlight the need to examine occupational sitting in a way that is cognisant of the potential multiple influences of sitting behaviour. Private offices are typically assigned to higher status employees or employees required to conduct more individualised job tasks. While our analyses adjusted for occupational level to minimise the impact of these issues, the associations between the duration and frequency of breaks in sitting and overall connectivity in counter expected directions suggest that these issues may have still confounded these associations and that more refined measures of job tasks should be used in future research.

Proximity of co-workers was positively associated with duration of sitting in open plan office types. This is both in agreement and in contrast to previous studies examining proximity and movement or face-to-face interaction [8,16,18]. Rashid and colleagues reported that closeness, a variable related to proximity, was inversely associated with frequency of face-to-face interactions and positively associated with sedentary workstations activities [8]. A potential explanation for this was mutual surveillance between co-workers [8], and this may partially explain the results observed in the current study. Alternatively, it may be that co-workers in very close proximity communicate without leaving their workstations; this is supported by evidence that conversation related distractions are frequent in open plan offices [47]. This issue should be explored further, as a threshold effect may occur as very proximally located employees can interact face-to-face without moving from their workstation, while after some (as yet unknown) distance face-to-face interaction requires one or all parties to move from their workstation. The way in which the breaks in sitting variable was operationalized may have also impacted the associations observed, as the interruptions to sitting driven by office spatial configuration may include breaks shorter than one minute in duration. The one minute threshold was used in this study to enable comparison to breaks in sedentary time measured by accelerometer [2] and to assist in recall. Since this item was developed, a measure of breaks in sitting without a minimum time threshold has been developed and tested [49], which may be more suited to understanding how office environments influence sitting behaviour. Research examining how spatial configuration impacts sitting behaviour using alternate measures of sitting behaviour and in more diverse populations will assist in clarifying the way in which office configuration influences sitting behaviours. Objective measures of sitting could be useful in further understanding these relationships as they will allow more detailed and accurate measures of sitting behaviour to be examined.

A key requirement in the development of the OFFESS was to take four spatial configuration characteristics of office environments (connectivity, integration, visibility of co-workers, and proximity of co-workers) that are related to occupant movement patterns as measured by Space Syntax methodologies and create four self-report scales that reflected each spatial configuration characteristic. These four Space Syntax measured constructs are frequently observed to be lower in Private Enclosed offices compared to Open Plan Offices [9,22], and the respective OFFESS scales, all displayed lower average scores in Private Enclosed offices compared to Open Plan Offices. The differences between OFFESS scales and office types were statistically significant with the exception of Overall Connectivity; however, in all instances differences were in the theoretically expected directions. Additionally, the internal consistency of individual OFFESS scales observed in the phase two population approached a level considered to be acceptable, providing some indication of the robustness and generalisability of the scales. So, while the criterion validity of the OFFESS was not examined, the pattern of differences between office types, associations between the OFFESS and sitting behaviours, and the level of internal consistency demonstrated in a separate sample provides some evidence of the construct validity. Comparison to a criterion measure of OFFESS constructs was not possible in the current study as detailed floor plans required for this analysis could not be obtained due to restricted access to some offices.

The OFFESS scales demonstrated substantial to excellent levels test-retest reliability (ICC = 0.70 - 0.87) indicating that its scales are stable over time; this is important for an instrument assessing environmental characteristics that are relatively static over time. The ICCs exhibited by the OFFESS are lower than those observed by the Perceived Workplace Environment Policy Scale (ICC = 0.97) that assessed individual, social, environmental, and policy-level characteristics of the workplace [50]. Although both the Perceived Workplace Environment Policy Scale and the OFFESS examine workplace environments, given the differences in the characteristics assessed by each of these instruments, the level of test-retest reliability between these instruments is not directly comparable. However, when comparing OFFESS to surveys that assess neighbourhood environments, examining similar spatial characteristics such as street connectivity, and service proximity, (e.g. access to shops), the OFFESS demonstrates higher levels of test-retest reliability than the Neighbourhood Environment Walkability Scale (ICC = 0.58 – 0.80) and the Physical Activity Neighbourhood Environment Survey (ICC = 0.64 – 0.84) [21,51]. This suggests that the OFFESS has levels of test-retest reliability comparable to other self-report instruments assessing related environmental characteristics.

Although the measurement properties of the OFFESS examined in the current study are encouraging, further examination of its measurement properties in larger samples and those working in varying office types would provide greater confidence in the measurement properties of the OFFESS. The classification of office types; used in the current study did not encompass offices that have a mixture of office types, for example, offices with open plan configurations in the centre and private or shared offices on the perimeter of the office. It is unknown how respondents of the current study who worked in this type of office responded to the item used to classify office type. Additionally, we acknowledge the time between repeat surveys in phase two was relatively small and future studies may seek to examine the reliability of the instrument over longer time periods and also its sensitivity to changes in the workplace environment. The OFFESS does not capture information on the unique characteristics of an individual workstation, such as seated, standing, or height adjustable; this is important to capture in future studies examining this topic as use of height adjustable workstations increases and is associated with reduced sitting [52]. Nor does the OFFESS capture information on the presence of destinations in the office environment (i.e. amenities, kitchens, toilets, cafeterias or meeting rooms). The availability, distance, and frequency of travel are factors that may impact upon how office destinations influence sitting behaviour. Greater insight is needed regarding the interrelationships of these factors to inform the development and implementation of such a measure.

The current study used an online administration of the instrument, yet we are confident that the instrument could be successfully administered in either paper-based or telephone-based surveys. Associations between sitting behaviours and the potential behavioural correlates should be examined within ecological models that are domain-specific [19,44]. Thus, a strength of the OFFESS is that it can be used to provide domain-specific associations between environments and behaviours for individual’s employed in office settings. Sitting time items were developed specifically for this study and demonstrated acceptable psychometric properties, and the level of test-retest reliability for the duration of sitting time item is superior to that compared to other measures of occupational sitting time [53]. The measure of breaks in sitting time used a different recall period (daily vs. hourly) compared to a similar measure [49], yet the frequency of breaks in sitting during the work day appears similar. Comparison of the sitting time measures used in this study to objective measures of sitting time (i.e. ActivPAL) or direct observation should be conducted to establish the validity of these measures.

## Conclusion

This study documented the development of the OFFESS to assess the spatial configuration of office environments. OFFESS scales have good levels of internal consistency, test-retest reliability and display some evidence of construct validity making it a useful instrument for this purpose. To better understand the role of office spatial configuration as an influence on sitting behaviour, assessment of spatial configuration should be conducted as part of a broader ecological model of behaviour. The overall length of the OFFESS is relatively short making it suitable for use in population based studies that seek to examine potential correlates of occupational sitting.

## Competing interests

The authors declare that they have no competing interests.

## Authors’ contributions

MJD was responsible for the overall conception and design of the manuscript, statistical analysis and interpretation of the data. MR and NC contributed to the initial conception of items and the critique of preliminary scales. All authors contributed to the drafting and critical revision of the manuscript. All authors read and approved the final manuscript.
